# LIFELONG LEARNING FOR THE OLDER POPULATION—TECHNOLOGY WITH THE OLDER LEARNERS

**DOI:** 10.1093/geroni/igad104.1163

**Published:** 2023-12-21

**Authors:** Yan-Jhu Su, Andrew Alberth

**Affiliations:** University of Massachusetts Boston, Boston, Massachusetts, United States; University of Massachusetts Boston, Boston, Massachusetts, United States

## Abstract

Advances in digital information technology and population aging are two hallmarks of the 21st century. Modern students are increasingly dependent on digital technology in their education. However, how to make digital technology less exclusive to young students remains an important issue. The COVID-19 pandemic has also popularized remote learning and the use of communication software among all ages. Teaching remotely has broadened access to education for all ages. Therefore, how to use technology to help older people achieve the goal of lifelong learning remains critical. This presentation will review the literature to explore the relationship between technology and older adults, as well as share the author’s experience leading older adults to use technology for online learning in Taiwan. The key takeaways from this presentation will help educators understand the benefits and challenges of technology use among older adults, and highlight ways to bridge the digital divide between older and younger learners in the classroom.

